# Plasma Amino Acids as Correlates of Serum Insulin-Like Growth Factor 1, Linear Growth, and Fat-Free Mass: A Cross-Sectional Study among Ugandan Children with Stunting

**DOI:** 10.1016/j.tjnut.2026.101543

**Published:** 2026-04-17

**Authors:** Anni Larnkjær, Joseph Mbabazi, Rolland Mutumba, Christian Ritz, Suzanne Filteau, André Briend, Kim F Michaelsen, Christian Mølgaard, Otto Savolainen, Ezekiel Mupere, Henrik Friis, Benedikte Grenov

**Affiliations:** 1 Department of Nutrition, Exercise and Sports, University of Copenhagen, Copenhagen, Denmark; 2 Department of Paediatrics and Child Health, School of Medicine, College of Health Sciences, Makerere University, Kampala, Uganda; 3 The National Institute of Public Health, University of Southern Denmark, Copenhagen, Denmark; 4 Department of Population Health, London School of Hygiene and Tropical Medicine, London, United Kingdom; 5 Tampere Center for Child, Adolescent and Maternal Health Research, Faculty of Medicine and Health Technology, Tampere University and Tampere University Hospital, Tampere, Finland; 6 Department of Biology and Biological Engineering, Chalmers University of Technology, Gothenburg, Sweden

**Keywords:** plasma amino acids, children, stunting, IGF-1, growth, animal-source foods, Uganda

## Abstract

**Background:**

Stunting is widespread in low-income settings because of inferior quality diets and infections, which downregulate serum concentration of insulin-like growth factor-1 (serum IGF-1). Associations among plasma concentrations of amino acids (plasma AA), serum IGF-1, and growth remain underexplored in humans.

**Objectives:**

The aim of this study was to assess the role of plasma AA as a correlate of serum IGF-1, linear growth, and fat-free mass (FFM) among stunted children.

**Methods:**

In a cross-sectional study nested in a nutrition intervention trial among 750 Ugandan children, aged 12–59 mo, with stunting, we assessed anthropometry, recent intake of animal-source foods (ASFs), fasting time, and body composition. Serum IGF-1, serum concentrations of the inflammatory markers C-reactive protein and α_1_-acid glycoprotein, and 21 plasma AA were measured.

**Results:**

Mean (standard deviation) age and height-for-age *z*-score (HAZ) were 32.0 (11.7) mo and −3.02 (0.74). Plasma AA data were available for 711−730 (95%–97%) children. For 18 AA, plasma concentrations were lower among children with elevated inflammatory markers. ASF intake within 24 h was associated with few AA; meat intake was associated with higher plasma methionine, whereas cow milk and egg intake were not associated with plasma concentration of any essential AA (EAA). Nearly all plasma AA, including all EAA, were positively associated with serum IGF-1, after adjusting for fasting and markers of inflammation. Only a few plasma AA, including methionine, threonine, and valine, were associated with FFM or FFM index. Only plasma tyrosine and taurine were associated with HAZ.

**Conclusions:**

Among stunted children, most plasma AA were positive correlates of serum IGF-1, but few correlated with HAZ or FFM. Further research is needed to clarify the role of plasma AA in the complex mechanisms underlying growth faltering in malnourished children.

## Introduction

Stunting is common among children in low-income settings and is associated with morbidity, mortality, and poor child development and educational achievement [1]. In addition, stunting is accompanied by deficits in fat-free mass (FFM) [2], which reduces insulin sensitivity, hence may increase the risk of cardiometabolic diseases later in life.

Low-quality diets and infectious diseases are the main causes of impaired growth in low-income settings [1]. In Africa, these diets are often based on maize and other starch-rich staple foods, with small amounts of legumes, fruits, and vegetables, and minimal or no animal-source foods (ASFs) [3,4]. Such diets have a high content of phytates, which impairs the digestibility and bioavailability of nutrients important for growth, such as zinc and proteins [4,5].

Protein is an important nutrient for growth [6]. Protein deficiency was previously considered to be the key nutritional deficiency contributing to childhood stunting, but later research focus shifted to deficiencies of zinc and other micronutrients [7]. However, although the protein intake may generally be adequate, it is possible that protein quality is not, and that low protein digestibility and bioavailability of essential amino acids (EAAs) contribute to impaired linear growth. A study from Malawi found that children with stunting had lower serum concentrations of all EAA [8] compared with nonstunted children. The authors suggested that lack of EAA in the diet contributed to stunting, and that the disappointing effects of micronutrient supplementation and small-quantity lipid-based nutrient supplements (LNSs) on child growth in some trials could be explained by lack of EAA in the diet [8].

However, stunting as a dichotomous variable is a crude outcome. Rather than reflecting the current linear growth process, it is a statistical construct based on an accumulation of effects of past exposures, and only partly reversible. Although change in height-for-age *z*-score (HAZ) could be used, an alternative and likely more sensitive outcome in studies of impaired linear growth may be serum concentrations of insulin-like growth factor 1 (serum IGF-1), a key regulator of growth in childhood. Growth hormone released from the pituitary gland stimulates secretion of IGF-1 from the liver for endocrine signaling [9]. As such, IGF-1 released to the circulation reaches receptors in the epiphyseal growth plates of bones, where it promotes chondrocyte differentiation, proliferation, and ossification, and thereby linear growth [10]. In addition to its hepatic production, IGF-1 is generated locally in muscle and bone, acting through paracrine and autocrine mechanisms [9].

Serum IGF-1 concentrations are known to decline in the presence of inflammation [11]. Deficiencies of zinc and other nutrients important for growth may also exert their growth-limiting effects through downregulation of serum IGF-1 concentrations. In a well-controlled study among rats, experimental zinc deficiency was shown to considerably reduce serum IGF-1 compared with pair-fed controls [12]. A trial among stunted children aged 4–36 mo in Vietnam found that zinc supplementation increased serum IGF-1 and linear growth [13]. In a large European trial, formula and follow-on formula with high compared with low protein content increased the plasma concentration of EAA and branched-chain amino acids (BCAAs), as well as serum IGF-1 [14].

**FIGURE 1:**
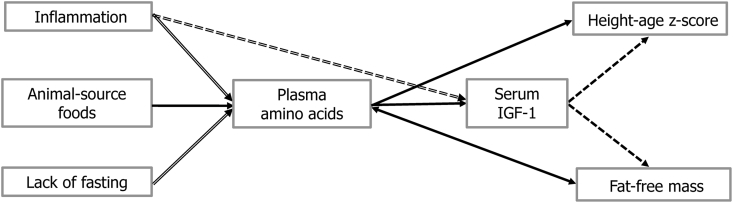
Conceptual framework illustrating factors influencing plasma amino acids and effects of plasma amino acids. Solid lines show associations investigated involving plasma amino acids, whereas dashed lines represent associations not involving plasma amino acids. Single lines denote positive influences, and double lines denote negative influences. IGF-1, insulin-like growth factor 1.

We have previously shown that serum IGF-1 was low among Ugandan children with stunting, partly because of inflammation, and that serum IGF-1 was positively associated with HAZ and FFM [15]. Furthermore, intake of cow milk, but not other ASF, was associated with higher serum IGF-1. On the basis of data from the same study, we aimed to assess the association between intake of ASF and plasma concentrations of amino acids (plasma AA). Moreover, we examined the associations between plasma AA and serum IGF-1, HAZ, and FFM (Figure 1). In addition, we investigated whether inflammation and fasting time were related to plasma AA concentrations.

## Methods

### Study design and ethics

The study used baseline data from the MAGNUS trial (Milk Affecting Growth, Cognition, and the Gut in Child Stunting), a randomized, controlled, 2 × 2 factorial trial investigating the effects of milk protein and whey permeate in LNS on growth and body composition of 750 Ugandan children with stunting. The MAGNUS trial was registered in the ISRCTN registry (ISRCTN13093195), and the protocol was approved by the School of Medicine Research Ethics Committee at Makerere University (REC REF 2019-013) and The Ugandan National Council of Science and Technology (SS 4927). Consultative approval was also obtained from the Danish National Committee on Biomedical Research Ethics (1906848). The study complied with the local guidelines for human studies and the principles of the Declaration of Helsinki. Written informed consent was obtained from all caregivers of the participating children before enrolment.

### Setting and participants

The study has been described in detail elsewhere [[16], [17], [18], [19]]. Briefly, participants were recruited in 2020 from the Jinja District, Eastern Uganda. Inclusion criteria were HAZ less than −2, aged 12–59 mo, and residence in the catchment area. Exclusion criteria included severe acute malnutrition, medical complications requiring hospitalization, peanut or milk allergies, disabilities that impeded the ability to eat or measure height, participation in other studies or plans to leave the catchment area within 6 mo. Furthermore, only 1 child per household was included.

### Data collection

Information on sociodemographics, current breastfeeding status, and any intake of cow milk, meat, or eggs within the last 24 h was collected by trained staff using interviewer-administered questionnaires. Information on the quantity of food intake was not available.

### Anthropometry and body composition

Trained staff measured height or length to the nearest 0.1 cm using an infant/child ShorrBoard (Weigh and Measure) and weight to the nearest 100 g using a digital double weighing scale (SECA 876). Mid-upper arm circumference was measured to the nearest 0.1 cm using a standard measuring tape (UNICEF SD) on the left arm. All anthropometric measurements were performed in triplicate, and the median value was used. BMI was calculated as body weight (kg) divided by squared length or height (m^2^). Age- and sex-specific anthropometric *z*-scores were calculated according to WHO growth standards using WHO Anthro software and STATA14 (Stata, College Station, Texas, USA) [16]. Bioelectrical impedance was measured twice using the BODYSTAT 500 device (Bodystat Ltd.) and the mean was used to estimate FFM by applying an age-, sex-, and stunting-appropriate equation developed for this population [20]. Fat mass (FM) was obtained by subtracting FFM from body weight. FM index and FFM index (FFMI) were calculated as FM or FFM (kg) divided by squared length or height (m^2^) [[21], [22], [23]].

### Blood samples and analyses

Venous blood samples of ≤6 mL were drawn into serum, lithium-heparin, and ethylenediaminetetraacetic blood collection tubes and processed as described previously [16,17]. The estimated fasting time was recorded by the staff. Samples were centrifuged at 2300 × *g* for 10 min and frozen at −20°C at a local laboratory. Weekly, samples were transferred to a biorepository and stored at −80°C until shipment on dry ice to Europe for analysis.

Malaria and hemoglobin concentrations were assessed from whole blood at the field laboratory using rapid diagnostic tests (SD Bioline Malaria Ag Pf; Abbott and Hb201; HemoCue, respectively). Serum C-reactive protein (CRP) and α_1_-acid glycoprotein (AGP) were used as fast- and slow-reacting markers of inflammation, respectively, and measured using a combined sandwich ELISA at the VitMin Lab [24]. Interassay and intra-assay coefficients of variation were 5%–14%. Serum IGF-1 was analyzed on Immulite2000 Analyzer (Siemens Healthcare, GmbH) at the University of Copenhagen with intra-assay and interassay coefficients of variation of 1.9%–4.2% and 4.2%–7.2%.

Plasma AA concentrations were measured by liquid chromatography tandem mass spectrometry using a commercially available amine-reactive isotope-coded tags (aTRAQ reagent) kit for Amino Acid Analysis of Physiological Fluids (AB Sciex) at Chalmers University of Technology [17]. Prepared samples were analyzed using an AB Sciex QTRAP 6500+ system (AB Sciex) with a Nexera UHPLC system (Shimadzu). Analytes were separated on a Waters BEH C18 column (150 × 2.1 mm, 1.7 μm), and the mass spectrometer was set to monitor the transitions presented in Supplemental Table1 with the following ion source parameters: CUR 30, CAD MED, IS 5500, TEM 500, GS1 60, and GS2 50 and compound parameters: DP 30, EP 10, CE 30, and CXP 5. Precision within and between batches was assessed using AA quality control samples, and precision results are presented in Supplemental Table 1.

In total, 21 plasma AA were measured: 8 EAA (histidine, isoleucine, leucine, methionine, phenylalanine, threonine, tryptophan, and valine), 5 semiessential (semi-EAA) (arginine, L-glutamine, glycine, proline, and tyrosine), 3 non-EAA (L-aspargine, glutamic acid, and serine), and 5 other AA (citrulline, ethanolamine, hydroxy-L-proline, ornithine, and taurine). Lysine, along with a few other standard proteinogenic AA, was excluded from the dataset because the measurement precision was not stable, most likely because of insufficient peak stability across the large number of samples and batches analyzed, which prevented reliable quantification. Total EAA, total semi-EAA, and BCAA (isoleucine, leucine, and valine) were calculated as the sum of the concentrations of the respective AA.

### Statistics

Descriptive statistics are presented as mean and SDs or median and IQRs for normally and nonnormally distributed continuous variables, respectively, whereas categorical variables are given as *n* (%). Normal probability plots were used to visually evaluate the distribution. Comparison between sex was performed by an independent *t* test or Mann–Whitney for plasma AA with normal or skewed distribution. As seen in our conceptual framework (Figure 1), fasting time, inflammation, and intake of ASF may affect plasma AA concentrations. The influence of fasting time and inflammation was investigated by comparing the AA concentrations in different fasting time categories, as well as in different categories of the inflammation markers serum CRP and AGP, using analysis of variance. We then estimated the associations between intake of ASF (cow milk, meat, and eggs) and plasma AA (as dependent variables), whereas adjusting for age, sex, and fasting time in the multiple linear regression analysis. Moreover, we estimated the associations between standardized plasma AA (plasma AA *z*-scores, as independent variables) and serum IGF-1, HAZ, FFM, and FM (as outcomes) in multiple linear regression models adjusted for age, sex, and fasting time. Models including adjustment of inflammatory markers serum CRP and AGP as continuous variables were also conducted. To explore the influence of serum CRP and AGP separately, models including either adjustment for serum CRP or AGP were fitted.

Model assumptions for regression models and the impact of outliers on estimates and significance were checked by residual plots and Cook’s distance, respectively. *P* values < 0.05 were considered significant, whereas *P* values 0.05 to <0.10 were considered trends. Data analyses were performed using IBM SPSS Statistics (version 29.0; IBM).

## Results

Of the 7611 children screened, 1112 were stunted and referred to the study sites for eligibility assessment of which 750 (67%) were enrolled [16]. The mean (SD) HAZ score was −3.02 (0.74) (Table 1), and 42% (*n* = 314) were severely stunted (HAZ < −3). Moderate wasting was observed in 5% (*n* = 39) of the children. The mean (SD) age was 32.0 (11.7) mo, 45% (*n* = 338) were girls, and 13% (*n* = 95) were still being breastfed. Approximately one third of the children had consumed cow milk or meat in the past 24 h, whereas only 8% (*n* = 59) of the children had eaten eggs.

**TABLE 1:** Characteristics of the 750 children with stunting

	n	
Age (mo)	750	32.0 (11.7)
12–23		222 (30%)
24–35		259 (34%)
36–60		269 (36%)
Sex (girls)	750	338 (45%)
Residence (urban)	750	415 (55%)
Household size	750	5 (4;7)
Maternal education (≥primary)	713	375 (53%)
Own livestock	750	379 (51%)
Anthropometry		
Weight (kg)	749	10.6 (2.0)
Length/height (cm)	750	81.7 (7.35)
Mid-upper arm circumference (cm)	750	14.2 (1.2)
Height-for-age *z*-score	750	−3.02 (0.74)
Height-for-age *z*-score <–3		314 (42%)
Weight-for-height *z*-score	749	−0.36 (0.99)
Body composition		
Fat mass (kg)	746	1.80 (0.88)
Fat-free mass (kg)	746	8.80 (1.45)
Fat mass index (kg/m^2^)	746	2.66 (1.16)
Fat-free mass index (kg/m^2^)	746	13.12 (0.070)
Blood analyses		
Hemoglobin (g/L)	743	103.8 (14.7)
Insulin-like growth factor 1 (ng/mL)	740	37.4 (24.1, 53.3)
C-reactive protein (mg/L)	741	1.57 (0.33, 8.29)
α_1_-acid glycoprotein (g/L)	741	1.29 (0.52)
Malaria test (positive)	737	292 (40%)
Diet		
Breastfed (currently)	746	95 (13%)
Intake of cow’s milk^1^	750	227 (30%)
Intake of meat^1^	750	287 (38%)
Intake of egg^1^	750	59 (8%)

Results are reported as *n* (%), mean (SD), or median (IQR).

1 Assessed by 24-h recall for intake of food groups.

### Plasma AA concentrations

Plasma AA were available for 95%–97% (*n* = 711–730) of the children (Table 2). Boys had lower concentrations of L-glutamine, hydroxy-L-proline, ornithine, and taurine (*P* ≤ 0.039), but there were no sex differences for the other AA.

**TABLE 2:** Plasma amino acids concentration (μM)

Amino acids	*n*	
Essential		
Histidine	725	76.4 (33.3)
Isoleucine	727	43.6 (33.5, 59.7)
Leucine	725	85.6 (30.7)
Methionine	723	17.0 (7.86)
Phenylalanine	726	49.8 (24.2)
Threonine	726	61.3 (30.0)
Tryptophan	711	26.9 (16.1, 38.5)
Valine	726	145 (67.2)
Semiessential		
Arginine	726	48.2 (35.5, 65.9)
L-glutamine	728	561 (248)
Glycine	726	258 (127)
Proline	726	175 (131, 242)
Tyrosine	726	48.3 (24.4)
Nonessential		
L-asparagine	729	55.5 (23.0)
Glutamic acid	724	72.7 (36.7)
Serine	730	130 (60.9)
Other		
Citrulline	724	23.0 (11.7)
Ethanolamine	728	10.3 (8.21, 12.9)
Hydroxy-L-proline	727	23.4 (7.5, 31.7)
Ornithine	722	80.8 (57.0, 116)
Taurine	728	69.9 (50.6, 92.0)
Essential	702	514 (215)
Semiessential	719	636 (281)
Branched chain	724	280 (125)

Results are shown as mean (SD) or median (IQR).

Fasting time before blood sampling was 0–2 h for 4.1% (*n* = 31), 2–5 h for 54.7% (*n* = 408), and >5 h for 41.2% (*n* = 307) of the children.

The plasma concentration of 5 AA (arginine, citrulline, ethanolamine, and ornithine) was higher with longer fasting time (Supplemental Table 2a).

### Inflammation as a correlate of plasma AA

Of the 21 AA, the plasma concentrations of 18 were inversely associated with serum CRP and/or AGP (Supplemental Table 2b and 2c). Only ethanolamine and ornithine were not associated with elevated AGP or CRP, and phenylalanine was higher with elevated CRP.

### Intake of ASF as correlates of plasma AA concentrations

Intake of cow milk within the last 24 h was associated with 29.0 μM [95% confidence interval (CI): 8.42, 49.5] lower plasma glycine and 7.73 μM (95% CI: 0.59, 14.9) lower plasma taurine after adjustment for age, sex, and fasting time (Table 3). Intake of meat was positively associated with 1.46 μM (95% CI: 0.26, 2.66), higher plasma methionine (EAA) and 3.66 μM (95% CI: 0.16, 7.15) higher L-asparagine (non-EAA). Likewise, meat was associated with higher plasma concentration of 2 other AA; hydroxy-L-proline (5.89 μM, 95% CI: 3.13, 8.65) and taurine (6.84 μM, 95% CI: 0.14, 13.5). Intake of eggs was not associated with any plasma AA.

**TABLE 3:** Associations between intake of cow milk, meat, or egg and plasma amino acids by linear regression analyses^1^

	Cow’s milk^2^			Meat^2^			Egg^2^		
	*β*	95% CI	*P*	*β*	95% CI	*P*	*β*	95% CI	*P*
Amino acids (μM)									
Essential									
Histidine	−4.64	−10.1, −0.78	0.093	3.92	−1.17, 9.00	0.13	−2.16	−11.4, 7.05	0.65
Isoleucine	−0.71	−5.30, 3.88	0.76	1.33	−2−97, 5.63	0.54	4.58	−3.24, 12.39	0.25
Leucine	0.34	−6.10, 6.89	0.90	3.55	−2.52, 9.61	0.25	−1.51	−12.54, 9.52	0.79
Methionine	0.72	−0.57, 2.00	0.27	1.46	0.26, 2.66	0.017	0.90	−1.29, 3.09	0.42
Phenylalanine	−2.36	−6.29, 1.57	0.24	−0.002	−3.69, 3.68	0.99	−4.19	−10.84, 2.47	0.22
Threonine	−3.01	−1.87. 7.80	0.23	3.72	−0.86, 8.29	0.11	1.56	−6.73, 9.85	0.71
Tryptophan	0.08	−5.87, 6.02	0.98	−0.45	−6.00, 5.11	0.88	−1.92	−11.95, 8.11	0.71
Valine	1.09	−9.83, 12.0	0.64	6.54	−3.68, 16.8	0.21	−0.75	−19.5, −18.0	0.94
Semiessential									
Arginine	−2.83	−7.67, 2.01	0.25	2.01	−2.53, 6.55	0.39	1.27	−6.95, 9.49	0.76
L-glutamine	−10.6	−50.7, 29.5	0.60	14.5	−23.1, 52.1	0.45	−12.1	−80.5, 56.2	0.73
Glycine	−29.0	−49.5, −8.42	0.006	12.8	−6.57, 32.1	0.20	−14.1	−49.3, 21.1	0.43
Proline	−6.50	−26.43, 13.43	0.52	5.80	−12.9, 24.5	0.54	−11.2	−45.1, 22.8	0.52
Tyrosine	2.14	−1.82, 6.10	0.29	2.74	−0.97, 6.45	0.15	−1.26	−7.98, 5.47	0.71
Nonessential									
L-asparagine	−2.03	−5.77, 1.70	0.29	3.66	0.16, 7.15	0.041	−2.54	−8.89, 3.81	0.43
Glutamic acid	−2.43	−8.41, 3.55	0.43	0.07	−5.54, 5.68	0.98	0.87	−9.27, 11.01	0.87
Serine	−9.63	−19.4, 0.17	0.054	5.83	−3.37, 15.0	0.21	1.63	−15.1, 18.3	0.85
Other									
Citrulline	−1.27	−3.16, 0.62	0.19	0.73	−1.05, 2.50	0.42	0.74	−2.47, 3.95	0.65
Ethanolamine	−0.25	−1.33, 0.83	0.65	−0.31	−1.32, 0.71	0.55	−1.12	−2.96, 0.72	0.23
Hydroxy-L-proline	−2.64	−5.61, 0.33	0.081	5.89	3.13, 8.65	<0.001	−0.84	−5.82, 4.34	0.78
Ornithine	−3.85	−16.15, 8.45	0.54	10.8	0.70, 22.3	0.066	−8.23	−29.3, 12.5	0.43
Taurine	−7.73	−14.9, −0.59	0.034	6.84	0.14, 13.5	0.045	2.36	−9.79, 14.5	0.70
Essential	−4.10	−40.0, 31.8	0.82	20.1	−13.2, 53.5	0.23	−5.58	−65.0, 54.7	0.86
Semiessential	−37.6	−83.6, 8.42	0.11	23.0	−20.1, 66.2	0.30	−26.4	−105, 51.8	0.51
Branched chain	0.67	−19.7, 21.1	0.95	11.5	−7.54, 30.6	0.24	1.85	−33.0, 36.7	0.92

1 Values are slope coefficients, 95% confidence interval (CI) and *P* values. *n =* 702–730.

2 Intake of food groups assessed by 24-h recall. Models are adjusted for age, sex, and fasting time.

### Plasma AA as correlates of IGF-1, HAZ, and body composition

In analyses adjusted for age, sex, and fasting time (Table 4), almost all plasma AA, including all the sum of EAA, semi-EAA, and BCAA, were positively associated with IGF-1 concentrations. For most AA, the slope coefficients corresponded to 1.3–4.6 ng/mL higher serum IGF-1 per *z*-score higher plasma AA. Adjustment for inflammation (model 2) slightly diminished the slope coefficients for most of the associations.

**TABLE 4:** Associations between plasma amino acids and serum insulin-like growth factor-1 by linear regressions analyses^1^

Amino acid *z*-score	Serum insulin-like growth factor-1 (ng/mL)					
	*β*	Model 1 95% CI	*P*	*β*	Model 2 95% CI	*P*
Essential						
Histidine	2.56	1.13, 3.96	<0.001	1.17	0.32, 3.03	0.016
Isoleucine	2.37	0.95, 3.79	0.001	1.58	0.23, 2.94	0.022
Leucine	2.91	1.49, 4.32	<0.001	2.09	0.74, 3.44	0.002
Methionine	2.64	1.22, 4.06	<0.001	1.63	0.27, 2.99	0.019
Phenylalanine	1.76	0.34, 3.19	0.015	1.94	0.58, 3.29	0.005
Threonine	4.16	2.77, 5.56	<0.001	2.84	1.48, 4.20	<0.001
Tryptophan	2.72	1.29, 4.15	<0.001	1.55	0.17, 2.92	0.027
Valine	4.64	3.24, 6.03	<0.001	3.19	1.82, 4.56	<0.001
Semiessential						
Arginine	3.23	1.80, 4.65	<0.001	2.04	0.67, 3.42	0.004
L-glutamine	2.47	1.04, 3.89	<0.001	1.24	−0.13, 2.62	0.076
Glycine	1.60	0.16, 3.03	0.030	1.72	0.35, 3.10	0.017
Proline	1.37	−0.056, 2.79	0.060	1.11	−0.30, 2,52	0.12
Tyrosine	4.64	3.25, 6.03	<0.001	3.23	1.86, 4.60	<0.001
Nonessential						
L-asparagine	2.23	0.80, 3.63	0.002	1.53	0.18, 2.89	0.027
Glutamic acid	2.91	1.49, 4.32	<0.001	2.69	1.35, 4.03	<0.001
Serine	1.37	−0.067, 2.80	0.062	0.86	−0.58, 2.30	0.24
Other						
Citrulline	3.64	2.22, 5.05	<0.001	2.28	0.89, 3.67	0.001
Ethanolamine	1.42	−0.036, 2.88	0.056	1.81	0.44, 3.18	0.010
Hydroxy-L-proline	4.04	2.64, 5.43	<0.001	2.91	1.56, 4.27	<0.001
Ornithine	1.75	0.30, 3.21	0.018	1.75	0.38, 3.12	0.012
Taurine	3.57	2.13, 5.01	<0.001	2.68	1.29, 4.07	<0.001
Essential	4.08	2.66, 5.51	<0.001	2.88	1.49, 4.26	<0.001
Semiessential	2.47	1.04, 3.89	<0.001	2.30	0.85, 3.66	<0.001
Branched chain	6.84	4.41, 9.26	<0.001	4.72	2.38, 7.06	<0.001

All models are adjusted for age, sex, and fasting time (model 1).

Model 2 is further adjusted for concentrations of C-reactive protein and α_1_-acid glycoprotein.

1 Values are slope coefficients, 95% confidence interval (CI) and *P* values. *n =* 699–730.

Only plasma tyrosine and taurine were positively associated with HAZ after adjusting for age, sex, and fasting time (Table 5). HAZ was 0.058 (95% CI: 0.005, 0.11) and 0.071 (95% CI: 0.018, 0.13) higher per *z*-score tyrosine and taurine, respectively. Plasma threonine, valine, tyrosine, hydroxy-L-proline and taurine as well as the sum of plasma EAA and BCAA, were positively associated with FFM, whereas plasma proline was negatively associated with FFM. The absolute values of slope coefficients were of the same order of magnitude (0.047–0.061 kg FM/plasma AA *z*-score); all adjusted for age, sex, and fasting time. When indexing for height (i.e., FFMI), only the association with plasma threonine remained. However, plasma methionine showed a positive association, and the slope coefficient was in the same order of magnitude. The impact of inflammation on the significant associations with HAZ, FFM, and FFMI, presented in Table 5, was assessed by further adjusting the model for both serum CRP and AGP (Supplemental Table 3). All associations disappeared except for 2: the associations between plasma proline and FFM and between plasma taurine and HAZ which remained but with diminished slope coefficients of 12% and 21%, respectively.

**TABLE 5:** Associations between plasma amino acids and height-for-age *z*-score, fat-free mass or fat-free mass index by linear regressions analyses^1^

Amino acid *z*-score	HAZ			FFM (kg)			FFMI (kg/m^2^)		
	*β*	95% CI	*P*	*β*	95% CI	*P*	*β*	95% CI	*P*
Essential									
Histidine	-−0.015	−0.069, 0.038	0.58	0.001	−0.038, 0.040	0.96	−0.016	−0.056, 0.024	0.46
Isoleucine	0.009	−0.062, 0.045	0.80	0.008	−0.032, 0.047	0.62	0.025	−0.016, 0.065	0.23
Leucine	0.020	−0.034, 0.074	0.46	0.023	−0.016, 0.062	0.25	0.009	−0.032, 0.049	0.68
Methionine	0.003	−0.051, 0.057	0.91	0.031	−0.008, 0.071	0.12	0.041	0.001, 0.081	0.047
Phenylalanine	−0.011	−0.065, 0.042	0.68	−0.001	−0.041, 0.038	0.95	−0.008	−0.048, 0.033	0.70
Threonine	0.035	−0.019, 0.088	0.20	0.048	0.000, 0.081	0.016	0.041	0.000, 0.081	0.048
Tryptophan	0.029	−0.024, 0.083	0.28	0.023	0.016, 0.063	0.25	0.017	−0.024, 0.057	0.42
Valine	0.048	−0.006, 0.10	0.079	0.060	0.021, 0.099	0.003	0.024	−0.017, 0.064	0.25
Semiessential									
Arginine	0.030	−0.024, 0.084	0.28	0.030	−0.009, 0.070	0.13	−0.001	−0.042, 0.040	0.96
L-glutamine	0.029	−0.025, 0.083	0.29	0.031	−0.009, 0.070	0.12	0.022	−0.018, 0.063	0.28
Glycine	−0.016	−0.069, 0.038	0.57	−0.014	−0.054, 0.025	0.47	−0.024	−0.065, 0.016	0.24
Proline	−0.047	−0.10, 0.006	0.082	−0.051	−0.090, −0.012	0.011	−0.017	−0.057, 0.023	0.40
Tyrosine	0.058	0.005, 0.11	0.033	0.050	0.011, 0.089	0.012	0.006	−0.034, 0.047	0.76
Nonessential									
L-asparagine	−0.003	−0.056, 0.051	0.92	−0.001	−0.040, 0.039	0.98	−0.007	−0.047, 0.033	0.74
Glutamic acid	−0.009	−0.063, 0.044	0.73	−0.005	−0.046, 0.033	0.76	−0.018	−0.059, 0.022	0.38
Serine	−0.025	−0.079, 0.028	0.36	−0.012	−0.051, 0.027	0.55	0.006	−0.035, 0.046	0.78
Other									
Citrulline	−0.013	−0.067, 0.041	0.63	0.020	−0.019, 0.060	0.32	0.039	−0.002, 0.080	0.061
Ethanolamine	−0.010	−0.063, 0.044	0.73	0.001	−0.039, 0.040	0.98	−0.023	−0.064, 0.017	0.26
Hydroxy-L-proline	0.042	−0.011, 0.096	0.12	0.061	0.022, 0.100	0.002	0.031	−0.009, 0.071	0.13
Ornithine	0.016	−0.039, 0.070	0.57	0.034	−0.005, 0.074	0.090	0.036	−0.005, 0.077	0.084
Taurine	0.071	0.018, 0.13	0.009	0.047	0.008, 0.086	0.019	−0.022	−0.063, 0.019	0.29
Essential	0.036	−0.018, 0.090	0.20	0.046	0.006, 0.085	0.024	0.018	−0.023, 0.060	0.38
Semiessential	−0.019	−0.072, 0.035	0.50	−0.022	−0.062, 0.017	0.27	−0.023	−0.063, 0.018	0.27
Branched chain	0.053	−0.04, 0.15	0.26	0.072	0.005, 0.14	0.036	0.037	−0.033, 0.11	0.30

Abbreviations: CI, confidence interval; FFM, fat-free mass; FFMI, fat -free mass index; HAZ, height-for-age *z*-score.

1 Values are slope coefficients, 95% CI and *P* values. *n* = 699-730. Models are adjusted for age, sex, and fasting time.

The individual impacts of the fast and slow-reacting inflammatory markers, serum CRP and AGP, respectively, were investigated using models adjusted separately for either serum CRP or AGP. In models adjusted for serum CRP, most associations remained despite reductions in slope coefficients (Supplemental Table 4).

## Discussion

We first examined the roles of fasting duration, inflammation, and intake of ASF as correlates of plasma AA concentrations. As we expected, a short interval since last eating and elevated inflammation markers were associated with lower plasma AA concentrations. Intake of cow milk the previous 24 h was associated with lower plasma glycine and taurine, whereas intake of meat was associated with higher plasma methionine, L-asparagine, hydroxy-L-proline, and taurine. Intake of egg was not associated with any plasma AA. We then investigated plasma AA as correlates of serum IGF-1, HAZ, FFM, and FFMI. Interestingly, the plasma concentrations of almost all AA were positively associated with serum IGF-1. These associations were not explained by confounding by inflammation. The plasma concentrations of a few AA, including methionine, threonine, and valine, were associated with FFM or FFMI, whereas tyrosine and taurine were associated with HAZ.

### Inflammation and plasma AA concentrations

Most plasma AA concentrations showed an inverse association with serum CRP and/or AGP except for plasma phenylalanine, ethanolamine, and ornithine. To the extent these associations are causal, they most likely reflect effects of inflammation on plasma AA, although the possibility cannot be excluded that the availability of specific AA may affect the inflammatory response. This could be explained by reduced appetite resulting in decreased dietary intake and impaired absorption of AA, as well as increased catabolism of AA in the gut and uptake and use of AA for protein synthesis in the liver. A lower concentration of the transport proteins may further contribute to these associations.

### Animal-source-foods and plasma AA concentrations

In contrast to our hypothesis, ASF was only associated with a few plasma AA. Meat intake was associated with higher plasma methionine, but not with other plasma EAA. Intake of cow’s and eggs was not associated with any plasma EAA. This may be surprising, because ASF are good sources of EAA, and because we previously found, based on data from the same study, that cow milk intake was associated with serum IGF-1 [15]. There may be several explanations for the lack of association between ASF and plasma AA. First, we only had data on whether the child had been fed cow milk, meat, and eggs the previous day, which may not mirror differences in habitual intake. Second, a considerable proportion of dietary intake of EAA and other AA is catabolized as part of the first-pass metabolism in the small intestines [25], to provide energy and as precursors for compounds (nucleotides, glutathione, nitric oxide, and citrulline, and other AA) important to the integrity of the intestinal mucosa [26,27]. Almost all glutamine, glutamate, and aspartate, and ≤50% of a range of other AA (arginine, proline, branched-chain: leucine, isoleucine, and valine; and methionine, lysine, phenylalanine, threonine, glycine, and serin) are, therefore, not available for extraintestinal tissues [27]. Of the AA that are not catabolized in the intestine, but released to the portal circulation, a large proportion is used by the liver to synthesize various peptides and proteins [28]. Therefore, the pattern of AA in the systemic circulation will be considerably different from the pattern of AA in the diet [27]. Yet, several studies have reported that protein and AA intake increase plasma AA concentrations and that the increase depends on the source, dose, and age of the consumer [[29], [30], [31], [32], [33]]. However, these studies were primarily conducted in healthy adults, with plasma AA concentrations measured relatively soon after protein intake but 1 study in healthy infants also demonstrated the influence of infant feeding on plasma AA showing that plasma EAA were higher in formula fed than in breastfed infants [33]. Nevertheless, the diet, absorption, and plasma AA concentrations in this study of stunted children may differ substantially, making the results difficult to compare. The few associations of ASF with the plasma concentration of other AA could be chance findings, considering the large number of tests carried out.

### Plasma AA and serum IGF-1

A study in rats showed that an AA-free diet reduced plasma IGF-1 compared with not only ad libitum fed, but also pair-fed controls (given same amount of food as the rats on an AA-free diet) [34]. Interestingly, diets deficient in only a single EAA (leucine, lysine, methionine, or threonine) also dramatically reduced plasma IGF-1, suggesting that ≥4 of the 8 AA are critical for IGF-1 synthesis. A randomized study in healthy infants investigated the influence on plasma AA and serum IGF-1 of 2 different formulas: 1 supplemented with specific free AA (tryptophan, phenylalanine) and a standard formula without free AA but with a higher overall protein content [33]. They found that serum IGF-1 was modulated by AA intake as formula supplemented with free individual AA resulted in higher IGF-1 concentrations than standard formula. Furthermore, the concentration of several AA had a direct effect on plasma IGF-1. In particular, tryptophan, threonine, and histidine showed an increase in plasma IGF-1. It is notable that threonine was also one of the AA essential for IGF-1 shown in the rat study above [34].

Overall, this is in line with our findings that plasma concentrations of all EAA and most non-EAA were positively associated with serum IGF-1, even after adjusting for inflammation markers.

### Plasma AA and linear growth, and FFM

Lower concentrations of all EAA among stunted compared with nonstunted children have been shown in Malawi [35]. In our study—among children with stunting—plasma methionine, threonine, and valine were the only EAA associated with FFM or FFMI. It is important to note that 2 of these, methionine and threonine, are among the 4 single-EAA-deficiencies shown to be essential to IGF-1 synthesis in the study by Takenaka et al. [34]. Only plasma concentrations of 2 non-EAA (tyrosine, taurine) were associated with HAZ in the present study. Nevertheless, almost all plasma AA were associated with serum IGF-1, which promotes bone growth and muscle mass [36]. This may suggest that serum IGF-1 may be a more sensitive marker of growth as height and body composition change slowly over time. However, it should be noted that the endocrine effect of IGF-1, produced in the liver and released to the circulation, depends on binding to IGF-binding proteins and uptake in target cells. Furthermore, IGF-1 is also produced in bones and muscle, where it acts locally [36].

Other studies investigating the association between circulating AA and growth have shown inconsistent results. In a study from Malawi, where 62% of the children aged 12–59 mo were stunted, and serum concentrations of 16 AA including 8 EAA, 4 semi-EAA, 3 non-EAA and citrulline, ornithine, and taurine were positively associated with HAZ [8]. However, the analyses were not adjusted for inflammation or fasting [8]. In Ethiopian children aged 6–35 mo, of whom 30% were stunted, serum concentrations of lysine and tryptophan were measured. The study did not find relationships between these serum AA and linear growth after adjusting for factors including inflammation [37]. In another study from Malawi, all serum concentrations of proteinogenic AA except cysteine were measured in infants aged 12-mo, where 27% were stunted. Plasma concentrations of 7 AA (4 EAA: isoleucine, threonine, tyrosine, valine, and 3 non-EAA: glycine, proline, and serine) were associated with length velocity *z*-score measured from 6–12 mo [38].

The studies differ in setting, study population, number of circulating AA measured, adjustment for inflammation and fasting status, and growth outcomes assessed making it difficult to compare. To our knowledge, no studies have investigated associations with FFM or FFMI. All studies included both stunted and nonstunted children, which provides a broader range in HAZ than in our study.

### Strengths and limitations

A major strength was the large sample size and almost complete data, which gives high precision of estimates and high statistical power to detect differences between groups, and low risk of biases. Other strengths include adjustment for fasting duration, and that the analyses were conducted with and without adjustments for fast and slow-reacting markers of inflammation. The main limitations of the study are the cross-sectional design, which does not allow us to make causal inferences, as well as the lack of data on plasma lysine and the high coefficient of variation of several AA. Another limitation is the lack of data on the quantity of intake of ASF. Residual confounding cannot be excluded. We did not adjust for multiplicity as this was an explorative study; hence, there is a risk of chance findings. Furthermore, the inclusion of only stunted children limits the generalizability of the findings.

In conclusion, most of the plasma AA were positively associated with serum IGF-1, even after adjustment for inflammation markers. In contrast, the plasma concentration of very few AA was correlated with HAZ or FFM. In addition, intake of ASF was not a correlate of plasma AA concentrations. Future studies including longitudinal measurement of diet, plasma AA, and growth are needed to better understand the role of AA in the complex mechanisms underlying growth-faltering children.

## Author contributions

The authors’ responsibilities were as follows – EM, HF, BG: designed the research; EM, JM, RM, HF, BG, OS: conducted the research; AL, HF, CR: analyzed data; AL, HF, BG, SF, AB, CM, KFM: interpreted the data; AL, HF: wrote the first draft; HF: had primary responsibility for final content; and all authors: read and approved the final manuscript.

## Data availability

Personal data may not be shared with other researchers without prior written consent from the relevant institutions and authorities, as required by the Ugandan Data Protection and Privacy Act and the European General Data Protection Regulation.

## Declaration of Generative AI and AI-assisted technologies in the writing process

The author(s) declare that no generative AI or AI-assisted technologies were used in the writing of this manuscript.

## Funding

This research was funded by Arla Food for Health (https://arlafoodforhealth.com). Arla Food for Health is a public–private research partnership between the University of Copenhagen, Aarhus University and the dairy company, Arla. Additional funds were obtained from the Danish Dairy Research Foundation (https://danishdairyboard.dk/research/ddrf), Augustinus Fonden (https://augustinusfonden.dk), Læge Sofus Carl Emil Friis og Hustru Olga Doris Friis’ Legat, and A. P. Møller Fonden til Lægevidenskabens Fremme (https://www.apmollerfonde.dk/ansoegning/fonden-til-laegevidenskabensfremme). The funding sources were not involved in study design, collection, analysis, interpretation, or decision to publish.

## Conflict of interest

The authors report no conflicts of interest.
