# Supplementary material for: Plasma Amino Acids as Correlates of Serum Insulin-Like Growth Factor 1, Linear Growth, and Fat-Free Mass: A Cross-Sectional Study among Ugandan Children with Stunting
Source: J Nutr. 2026 Apr 17;156(6):101543. doi: 10.1016/j.tjnut.2026.101543 (PMC13279294; doi:10.1016/j.tjnut.2026.101543)
Supplement: Multimedia component 1 [file mmc1.docx]

**Supplementary Table 1. Between batch coefficient of variation (CV%) from quality control (QC) replicates and MRM precursor product ion transitions for the monitored amino acid analytes.**

| **Amino acids** | **n** | **CV** | **QC1 m/z** | **QC3 m/z** |
| --- | --- | --- | --- | --- |
| Essential |  |  |  |  |
| Histidine | 725 | 16% | 304,2 | 121,1 |
| Isoleucine | 727 | 28% | 280,2 | 121,1 |
| Leucine | 725 | 8% | 280,2 | 121,1 |
| Methionine | 723 | 22% | 298,2 | 121,1 |
| Phenylalanine | 726 | 13% | 314,2 | 121,1 |
| Threonine | 726 | 18% | 268,2 | 121,1 |
| Tryptophan | 711 | 13% | 353,2 | 121,1 |
| Valine | 726 | 14% | 266,2 | 121,1 |
| Semi-essential |  |  |  |  |
| Arginine | 726 | 27% | 323,2 | 121,1 |
| L-glutamine | 728 | 16% | 295,2 | 121,1 |
| Glycine | 726 | 19% | 224,1 | 121,1 |
| Proline | 726 | 16% | 264,2 | 121,1 |
| Tyrosine | 726 | 13% | 330,2 | 121,1 |
| Non-essential |  |  |  |  |
| L-asparagine | 729 | 17% | 281,2 | 121,1 |
| Glutamic acid | 724 | 14% | 296,2 | 121,1 |
| Serine | 730 | 17% | 254,2 | 121,1 |
| Other |  |  |  |  |
| Citrulline | 724 | 12% | 324,2 | 121,1 |
| Ethanolamine | 728 | 14% | 210,2 | 121,1 |
| Hydroxy-L-proline | 727 | 13% | 280,1 | 121,1 |
| Ornithine | 722 | 18% | 429,3 | 121,1 |
| Taurine | 728 | 14% | 274,1 | 121,1 |

|  |  |  |  |
| --- | --- | --- | --- |
|  |  |  |  |

**Supplementary Table 2a. Plasma amino acids concentration according to fasting time**

|  | Fasting 0.5-2 hour | | | |  | Fasting >2-5 hour | | | |  | Fasting >5 hour | | | |  | p-value^1^ | |
| --- | --- | --- | --- | --- | --- | --- | --- | --- | --- | --- | --- | --- | --- | --- | --- | --- | --- |
| Amino acids (μM) | n | Mean | | 95% CI |  | n | Mean | 95% CI |  | | n | Mean | | 95% CI |  | |  |
| Essential |  | |  |  |  |  |  |  |  | |  | |  |  |  |  | |
| Histidine | 29 | | 71.2 | 61.7, 80.7 |  | 402 | 76.0 | 72.9, 79.1 |  | | 294 | | 77.5 | 73.4, 81.7 |  | 0.58 | |
| Isoleucine | 30 | | 49.5 | 38.0, 61.1 |  | 402 | 49.8 | 47.0, 52.7 |  | | 295 | | 50.5 | 47.4, 53.5 |  | 0.95 | |
| Leucine | 30 | | 81.7 | 68.8, 94.5 |  | 400 | 86.6 | 82.4, 90.8 |  | | 295 | | 84.3 | 80.2, 88.5 |  | 0.66 | |
| Methionine | 29 | | 17.7 | 14.5, 20.8 |  | 401 | 16.8 | 16.0, 17.6 |  | | 293 | | 17.1 | 16.3, 18.0 |  | 0.78 | |
| Phenylalanine | 29 | | 46.5 | 40.0, 53.0 |  | 402 | 51.2 | 48.8, 53.7 |  | | 295 | | 48.1 | 45.4.50.8 |  | 0.19 | |
| Threonine | 29 | | 60.9 | 49.5, 72.3 |  | 402 | 60.8 | 58.0, 63.6 |  | | 295 | | 61.9 | 58.3, 65.6 |  | 0.88 | |
| Tryptophan | 28 | | 33.4 | 24.7, 42.2 |  | 392 | 32.8 | 28.5, 37.1 |  | | 291 | | 30.5 | 27.5, 33.4 |  | 0.69 | |
| Valine | 30 | | 143 | 120, 166 |  | 402 | 141 | 135, 147 |  | | 294 | | 150 | 142, 158 |  | 0.21 | |
| Semi-essential |  | |  |  |  |  |  |  |  | |  | |  |  |  |  | |
| Arginine | 29 | | 52.6 | 42.4, 62.7 |  | 402 | 50.0 | 47.2, 52.8 |  | | 295 | | 60.2 | 56.6, 63.8 |  | **<0.001** | |
| L-glutamine | 30 | | 519 | 452, 586 |  | 402 | 548 | 524, 571 |  | | 296 | | 583 | 553, 614 |  | 0.11 | |
| Glycine | 30 | | 232 | 194, 270 |  | 402 | 261 | 250, 272 |  | | 294 | | 257 | 240, 274 |  | 0.45 | |
| Proline | 29 | | 207 | 170, 243 |  | 401 | 210 | 197, 222 |  | | 296 | | 191 | 177, 204 |  | 0.12 | |
| Tyrosine | 29 | | 50.3 | 41.5, 59.2 |  | 402 | 48.4 | 45.9, 50.8 |  | | 295 | | 47.9 | 45.2, 50.6 |  | 0.87 | |
| Non-essential |  | |  |  |  |  |  |  |  | |  | |  |  |  |  | |
| L-asparagine | 31 | | 58.3 | 50.1, 66.6 |  | 402 | 56.0 | 53.7, 58.3 |  | | 296 | | 54.5 | 51.9, 57.1 |  | 0.55 | |
| Glutamic acid | 28 | | 71 | 55.8, 86.2 |  | 401 | 72.2 | 68.5, 76.0 |  | | 295 | | 73.5 | 69.5, 77.5 |  | 0.88 | |
| Serine | 31 | | 128 | 111, 146 |  | 403 | 125 | 120, 130 |  | | 296 | | 136 | 127, 144 |  | 0.086 | |
| Other |  | |  |  |  |  |  |  |  | |  | |  |  |  |  | |
| Citrulline | 28 | | 19.7 | 16.4, 23.0 |  | 401 | 22.3 | 21.2, 23.3 |  | | 295 | | 24.2 | 22.7, 25.7 |  | **0.032** | |
| Ethanolamine | 30 | | 11.3 | 7.08, 15.4 |  | 402 | 12.4 | 11.7, 13.1 |  | | 296 | | 11.0 | 10.5, 11.6 |  | **0.028** | |
| Hydroxy-L-proline | 30 | | 23.8 | 19.7, 27.9 |  | 401 | 26.2 | 24.3, 28.2 |  | | 296 | | 27.2 | 25.2, 29.1 |  | 0.57 | |
| Ornithine | 28 | | 75.3 | 57.2, 93.3 |  | 401 | 92.9 | 87.4, 98.5 |  | | 293 | | 116 | 105, 128 |  | **<0.001** | |
| Taurine | 31 | | 72.4 | 61.5, 83.3 |  | 402 | 74.1 | 69.7, 78.6 |  | | 295 | | 83.4 | 78.5, 88.4 |  | **0.018** | |
| Essential | 27 | | 494 | 419, 568 |  | 386 | 511 | 489, 532 |  | | 289 | | 521 | 496, 547 |  | 0.72 | |
| Semi-essential | 27 | | 599 | 530, 668 |  | 399 | 642 | 615, 669 |  | | 293 | | 630 | 596, 664 |  | 0.68 | |
| Branched chain | 30 | | 274 | 231, 317 |  | 400 | 277 | 265, 290 |  | | 294 | | 285 | 270, 299 |  | 0.72 | |

^1^p-value: difference between fasting groups by ANOVA

**Supplementary Table 2b. Plasma amino acids concentration according to α_1_-acid glycoprotein (AGP)**

|  | AGP <0.8 g/L | | | |  | AGP 0.8-1.2 g/L | | | |  | | AGP >1.2 g/L | | |  | | p-value^1^ |
| --- | --- | --- | --- | --- | --- | --- | --- | --- | --- | --- | --- | --- | --- | --- | --- | --- | --- |
| Amino acids (μM) | n | | Mean | 95% CI |  | n | Mean | 95% CI | |  | | n | Mean | 95% CI |  | |  |
| Essential |  |  | |  |  |  |  | |  | |  |  |  |  | |  |  |
| Histidine | 127 | 84.9 | | 78.3, 91.5 |  | 237 | 77.5 | | 73.5, 81.4 | |  | 355 | 72.9 | 69.5, 76.4 | |  | **0.002** |
| Isoleucine | 126 | 58.6 | | 52.7, 64.5 |  | 237 | 49.1 | | 46.0, 52.2 | |  | 358 | 47.7 | 44.8, 50.7 | |  | **<0.001** |
| Leucine | 126 | 94.0 | | 87.3, 101 |  | 237 | 86.0 | | 81.0, 91.0 | |  | 356 | 82.3 | 78.1, 86.5 | |  | **0.017** |
| Methionine | 126 | 19.9 | | 18.3, 21.5 |  | 235 | 17.1 | | 16.0, 18.1 | |  | 356 | 15.9 | 15.2, 16.6 | |  | **<0.001** |
| Phenylalanine | 126 | 50.8 | | 46.6, 55.0 |  | 237 | 47.3 | | 44.8, 49.8 | |  | 357 | 51.2 | 48.3, 54.0 | |  | 0.14 |
| Threonine | 127 | 74.7 | | 68.2, 81.3 |  | 237 | 61.5 | | 57.9, 65.2 | |  | 356 | 56.3 | 53.5, 59.1 | |  | **<0.001** |
| Tryptophan | 125 | 42.6 | | 36.4, 48.8 |  | 232 | 33.5 | | 28.6, 38.3 | |  | 348 | 27.0 | 23.3, 30.6 | |  | **<0.001** |
| Valine | 126 | 175 | | 160, 190 |  | 236 | 146 | | 138, 153 | |  | 358 | 133 | 127, 140 | |  | **<0.001** |
| Semi-essential |  |  | |  |  |  |  | |  | |  |  |  |  | |  |  |
| Arginine | 127 | 63.0 | | 57.3, 68.6 |  | 237 | 55.3 | | 51.8, 58.8 | |  | 356 | 50.2 | 47.1, 53.4 | |  | **<0.001** |
| L-glutamine | 127 | 623 | | 577, 670 |  | 237 | 579 | | 551, 606 | |  | 358 | 527 | 501, 554 | |  | **<0.001** |
| Glycine | 126 | 263 | | 241, 285 |  | 237 | 254 | | 241, 266 | |  | 357 | 260 | 245, 275 | |  | 0.76 |
| Proline | 127 | 202 | | 185, 220 |  | 237 | 198 | | 185, 212 | |  | 356 | 204 | 190, 219 | |  | 0.84 |
| Tyrosine | 127 | 58.4 | | 53.7, 63.1 |  | 237 | 49.0 | | 46.1, 51.8 | |  | 356 | 44.2 | 41.8, 46.7 | |  | **<0.001** |
| Non-essential |  |  | |  |  |  |  | |  | |  |  |  |  | |  |  |
| L-asparagine | 128 | 61.3 | | 56.6, 66.6 |  | 237 | 54.7 | | 52.1, 57.3 | |  | 358 | 53.9 | 51.5, 56.3 | |  | **0.006** |
| Glutamic acid | 127 | 76.3 | | 69.4, 83.1 |  | 237 | 72.6 | | 67.6, 77.6 | |  | 354 | 71.5 | 67.9, 75.1 | |  | 0.46 |
| Serine | 129 | 140 | | 131, 149 |  | 237 | 129 | | 123, 135 | |  | 358 | 126 | 119, 134 | |  | 0.096 |
| Other |  |  | |  |  |  |  | |  | |  |  |  |  | |  |  |
| Citrulline | 127 | 27.4 | | 25.1, 29.8 |  | 237 | 24.1 | | 22.8, 25.4 | |  | 354 | 20.7 | 19.5, 21.9 | |  | **<0.001** |
| Ethanolamine | 127 | 11.4 | | 10.3, 12.5 |  | 237 | 11.6 | | 10.8, 12.4 | |  | 358 | 12.1 | 11.3, 12.8 | |  | 0.53 |
| Hydroxy-L-proline | 127 | 31.8 | | 28.2, 35.3 |  | 237 | 27.3 | | 25.6, 29.0 | |  | 357 | 24.0 | 22.0, 26.1 | |  | **<0.001** |
| Ornithine | 125 | 111 | | 96.8, 124 |  | 237 | 101 | | 91.4, 111 | |  | 354 | 99.0 | 91.0, 107 | |  | 0.34 |
| Taurine | 128 | 85.1 | | 77.3, 92.3 |  | 236 | 84.3 | | 78.3, 90.4 | |  | 358 | 71.1 | 66.8, 75.3 | |  | **<0.001** |
| Essential | 121 | 599 | | 553, 646 |  | 231 | 515 | | 490, 539 | |  | 344 | 485 | 463, 507 | |  | **<0.001** |
| Semi-essential | 123 | 663 | | 613, 713 |  | 237 | 629 | | 597, 660 | |  | 353 | 631 | 599, 663 | |  | 0.50 |
| Branched chain | 126 | 327 | | 302, 353 |  | 236 | 281 | | 266, 296 | |  | 356 | 263 | 251, 276 | |  | **<0.001** |

^1^Difference between AGP groups by ANOVA

**Supplementary Table 2c. Plasma amino acids concentration according to C-reactive protein (CRP)**

|  | CRP <2 mg/L | | |  | CRP 2-<5 mg/L | | | |  | CRP 5-<10 mg/L | | | |  | CRP 10-<15 mg/L | | | |  | CRP >15 mg/L | | | |  | p-value^1^ |
| --- | --- | --- | --- | --- | --- | --- | --- | --- | --- | --- | --- | --- | --- | --- | --- | --- | --- | --- | --- | --- | --- | --- | --- | --- | --- |
| Amino acids (μM) | n | Mean | 95% CI |  | n | Mean | 95% CI |  | | n | Mean | 95% CI |  | | n | Mean | 95% CI |  | | n | Mean | 95% CI |  | |  |
| Essential |  |  |  |  |  |  |  |  | |  |  |  |  | |  |  |  |  | |  |  |  |  | |  |
| Histidine | 384 | 82.2 | 78.9, 85.5 |  | 89 | 71.6 | 66.4, 76.9 |  | | 86 | 74.6 | 65.3, 83.8 |  | | 35 | 66.6 | 53.9, 79.2 |  | | 125 | 66.8 | 62.0, 71.5 |  | | **<0.001** |
| Isoleucine | 383 | 52.6 | 49.7, 55.6 |  | 91 | 49.0 | 44.5, 53.6 |  | | 87 | 50.6 | 44.1, 57.1 |  | | 35 | 44.0 | 34.0, 53.9 |  | | 125 | 44.3 | 39.8, 48.8 |  | | **0.038** |
| Leucine | 383 | 87.1 | 83.1, 91.1 |  | 90 | 85.4 | 79.2, 91.6 |  | | 87 | 84.0 | 75.6, 92.2 |  | | 35 | 81.1 | 59.9, 102 |  | | 124 | 83.2 | 76.2, 90.2 |  | | 0.81 |
| Methionine | 382 | 18.1 | 17.3, 18.9 |  | 90 | 15.9 | 14.7, 17.1 |  | | 85 | 17.5 | 15.5, 19.5 |  | | 35 | 14.7 | 11.7, 17.6 |  | | 125 | 14.6 | 13.5, 15.7 |  | | **<0.001** |
| Phenylalanine | 383 | 48.8 | 46.5, 51.0 |  | 90 | 48.3 | 44.3, 52.2 |  | | 86 | 48.0 | 41.9, 54.1 |  | | 35 | 48.4 | 38.8, 57.9 |  | | 126 | 55.8 | 50.9, 60.8 |  | | 0.050 |
| Threonine | 384 | 65.9 | 62.7, 69.2 |  | 90 | 61.3 | 55.6, 67.0 |  | | 86 | 59.5 | 53.4, 65.6 |  | | 35 | 49.4 | 38.8, 60.0 |  | | 125 | 51.6 | 47.7, 55.6 |  | | **<0.001** |
| Tryptophan | 375 | 35.5 | 31.7, 39.2 |  | 90 | 28.6 | 24.3, 32.8 |  | | 83 | 28.2 | 24.2, 32.1 |  | | 34 | 24.7 | 16.9, 32.4 |  | | 123 | 28.0 | 18.7, 37.2 |  | | 0.086 |
| Valine | 382 | 152 | 145, 160 |  | 91 | 140 | 129, 150 |  | | 87 | 136 | 122, 150 |  | | 35 | 126 | 101, 151 |  | | 125 | 136 | 126, 147 |  | | **0.018** |
| Semi-essential |  |  |  |  |  |  |  |  | |  |  |  |  | |  |  |  |  | |  |  |  |  | |  |
| Arginine | 384 | 58.1 | 55.0, 61.1 |  | 90 | 52.1 | 46.9, 57.3 |  | | 86 | 54.9 | 47.4, 62.3 |  | | 35 | 42.5 | 34.6, 50.5 |  | | 125 | 46.3 | 41.2, 51.4 |  | | **<0.001** |
| L-glutamine | 384 | 594 | 570, 619 |  | 91 | 544 | 504, 584 |  | | 87 | 529 | 470, 589 |  | | 35 | 502 | 411, 593 |  | | 125 | 510 | 465, 556 |  | | **0.003** |
| Glycine | 383 | 273 | 261, 285 |  | 91 | 267 | 229, 306 |  | | 86 | 256 | 227, 285 |  | | 35 | 216 | 177, 256 |  | | 125 | 220 | 203, 236 |  | | **<0.001** |
| Proline | 384 | 213 | 201, 225 |  | 91 | 198 | 179, 217 |  | | 86 | 200 | 163, 237 |  | | 35 | 173 | 134, 212 |  | | 124 | 179 | 161, 198 |  | | 0.055 |
| Tyrosine | 384 | 50.3 | 47.8, 52.8 |  | 90 | 45.7 | 42.0, 49.3 |  | | 86 | 46.8 | 41.4, 52.2 |  | | 35 | 43.4 | 32.5, 54.4 |  | | 125 | 46.3 | 42.2, 50.5 |  | | 0.18 |
| Non-essential |  |  |  |  |  |  |  |  | |  |  |  |  | |  |  |  |  | |  |  |  |  | |  |
| L-asparagine | 385 | 58.9 | 56.6, 61.3 |  | 91 | 52.6 | 48.7, 56.5 |  | | 87 | 54.7 | 48.9, 60.5 |  | | 35 | 49.0 | 41.9, 56.1 |  | | 125 | 49.3 | 45.6, 53.1 |  | | **<0.001** |
| Glutamic acid | 384 | 75.9 | 71.9, 79.8 |  | 90 | 75.6 | 68.5, 82.6 |  | | 85 | 73.4 | 65.8, 81.0 |  | | 35 | 65.0 | 54.9, 75.1 |  | | 124 | 62.6 | 57.0, 68.2 |  | | **0.006** |
| Serine | 386 | 138 | 133, 144 |  | 91 | 129 | 122, 137 |  | | 87 | 131 | 108, 154 |  | | 35 | 112 | 96.0, 128 |  | | 125 | 108 | 100, 116 |  | | **<0.001** |
| Others |  |  |  |  |  |  |  |  | |  |  |  |  | |  |  |  |  | |  |  |  |  | |  |
| Citrulline | 384 | 25.4 | 24.2, 26.5 |  | 90 | 21.9 | 20.1, 23.8 |  | | 85 | 22.3 | 19.1, 25.6 |  | | 35 | 18.4 | 14.3, 22.4 |  | | 124 | 18.2 | 16.7, 19.7 |  | | **<0.001** |
| Ethanolamine | 384 | 11.9 | 11.2, 12.6 |  | 91 | 11.7 | 10.4, 13.0 |  | | 87 | 12.3 | 10.4, 14.1 |  | | 35 | 10.6 | 8.90, 12.2 |  | | 125 | 11.7 | 10.7, 12.8 |  | | 0.79 |
| Hydroxy-L-proline | 384 | 29.7 | 27.5, 31.8 |  | 91 | 25.1 | 22.5, 27.7 |  | | 87 | 22.8 | 20.2, 25.3 |  | | 35 | 20.7 | 15.4, 25.9 |  | | 124 | 21.9 | 19.9, 23.9 |  | | **<0.001** |
| Ornithine | 382 | 105 | 97.4, 113 |  | 90 | 104 | 86.8, 121 |  | | 86 | 107 | 88.5, 125 |  | | 35 | 80.0 | 61.5, 98.4 |  | | 123 | 92.2 | 79.6, 105 |  | | 0.21 |
| Taurine | 385 | 86.5 | 81.5, 91.6 |  | 90 | 77.0 | 68.9, 85.0 |  | | 87 | 71.5 | 65.0, 78.0 |  | | 35 | 67.3 | 56.4, 78.2 |  | | 125 | 59.3 | 53.8, 64.8 |  | | **<0.001** |
| Essential | 371 | 539 | 516, 562 |  | 88 | 500 | 466, 534 |  | | 82 | 495 | 445, 545 |  | | 34 | 460 | 373, 546 |  | | 121 | 480 | 445, 515 |  | | **0.023** |
| Semi-essential | 380 | 671 | 643, 699 |  | 90 | 639 | 582, 696 |  | | 84 | 632 | 558, 706 |  | | 35 | 541 | 449, 633 |  | | 124 | 556 | 514, 599 |  | | **<0.001** |
| Branched chain | 382 | 292 | 279, 305 |  | 90 | 274 | 254, 294 |  | | 87 | 270 | 243, 297 |  | | 35 | 251 | 198, 304 |  | | 124 | 264 | 244, 283 |  | | 0.079 |

^1^Difference between CRP groups by one-way analysis of variance

**Supplementary Table 3.**  **Associations between plasma amino acids and serum insulin-like growth factor-I (IGF-I), height for age (HAZ), fat free mass (FFM) or fat free mass index (FFMI) adjusted for inflammation markers by linear regressions analyses^1^.**

| **Significant associations from Table 3** | |  | **Model 1^b^** | | |  | **Model 2** | | |  |
| --- | --- | --- | --- | --- | --- | --- | --- | --- | --- | --- |
| **Amino acids z-score** | **Independent variable** |  | β | 95 % CI | p |  | β | 95 % CI | p |  |
| Essential |  |  |  |  |  |  |  |  |  |  |
| Methionine | FFMI (kg/m^2^) |  | 0.041 | 0.001, 0.081 | **0.047** |  | 0.035 | -0.006, 0.075 | 0.091 |  |
| Threonine | FFM (kg) |  | 0.048 | 0.000, 0.081 | **0.016** |  | 0.015 | -0.023, 0.052 | 0.44 |  |
| Threonine | FFMI (kg/m^2^) |  | 0.048 | 0.000, 0.081 | **0.048** |  | 0.031 | -0.009, 0.072 | 0.13 |  |
| Valine | FFM (kg) |  | 0.060 | 0.021, 0.099 | **0.003** |  | 0.017 | -0.020, 0.055 | 0.37 |  |
| Semi-essential |  |  |  |  |  |  |  |  |  |  |
| Proline | FFM (kg) |  | -0.051 | -0.090, -0.012 | **0.011** |  | -0.045 | -0.082, -0.008 | **0.017** |  |
| Tyrosine | HAZ |  | 0.058 | 0.005, 0.11 | **0.033** |  | 0.015 | -0.039, 0.069 | 0.59 |  |
| Tyrosine | FFM (kg) |  | 0.050 | 0.011, 0.089 | **0.012** |  | 0.002 | -0.036, 0.040 | 0.91 |  |
| Other |  |  |  |  |  |  |  |  |  |  |
| Hydroxy-L-proline | FFM (kg) |  | 0.061 | 0.022, 0.100 | **0.002** |  | 0.033 | -0.004, 0.071 | 0.081 |  |
| Taurine | HAZ |  | 0.071 | 0.018, 0.13 | **0.009** |  | 0.056 | 0.003, 0.11 | **0.039** |  |
| Taurine | FFM (kg) |  | 0.047 | 0.008, 0.086 | **0.019** |  | 0.030 | -0.008, 0.684 | 0.12 |  |
| Essential | FFM (kg) |  | 0.046 | 0.006, 0.085 | **0.024** |  | 0.013 | -0.025, 0.051 | 0.49 |  |
| Branched chain | FFM (kg) |  | 0.072 | 0.005, 0.14 | **0.036** |  | 0.013 | -0.051, 0.078 | 0.69 |  |

^1^Values are slope coefficients, 95%CI, p-values. *n=*699-730*.* ^b^ Model 1 is adjusted for age, sex and fasting time. Model 2 is further adjusted for C-reactive protein and α_1_-acid glycoprotein concentrations.

**Supplementary Table 4. Associations between plasma amino acids and serum insulin-like growth factor-I (IGF-I), height for age (HAZ), fat free mass (FFM) or fat free mass index (FFMI) adjusted for reactive protein C (CRP) or α_1_-acid glycoprotein (AGP) concentrations by linear regressions analyses^1^.**

| **Significant associations from Table 3** | |  | **Model 1^b^** | | | |  | | **Model 1 and CRP** | | | |  | | **Model 1 and AGP** | | | |
| --- | --- | --- | --- | --- | --- | --- | --- | --- | --- | --- | --- | --- | --- | --- | --- | --- | --- | --- |
| **Amino acids z-score** | **Independent variable** |  | β | 95 % CI | p |  | | β | | 95 % CI | p |  | | β | | 95 % CI | p |  |
| Essential |  |  |  |  |  |  | |  | |  |  |  | |  | |  |  |  |
| Histidine | IGF-I (ng/ml) |  | 2.56 | 1.13, 3.96 | **<0.001** |  | | 2.08 | | 0.65, 3,50 | **0.004** |  | | 1.58 | | 0.23, 2.93 | **0.022** |  |
| Isoleucine | IGF-I (ng/ml) |  | 2.37 | 0.95, 3.79 | **0.001** |  | | 2.02 | | 0.60, 3.44 | **0.005** |  | | 1.54 | | 0.19, 2.90 | **0.026** |  |
| Leucine | IGF-I (ng/ml) |  | 2.91 | 1.49, 4.32 | **<0.001** |  | | 2.75 | | 1.34, 4.16 | **<0.001** |  | | 2.11 | | 0.77, 3.46 | **0.002** |  |
| Methionine | FFMI (kg/m^2^) |  | 0.041 | 0.001, 0.081 | **0.047** |  | | 0.044 | | 0.003, 0.085 | **0.034** |  | | 0.030 | | -0.011, 0.070 | 0.151 |  |
| Methionine | IGF-I (ng/ml) |  | 2.64 | 1.22, 4.06 | **<0.001** |  | | 2.20 | | 0.77, 3.62 | **0.003** |  | | 1.57 | | 0.21, 2.93 | **0.023** |  |
| Phenylalanine | IGF-I (ng/ml) |  | 1.76 | 0.34, 3.19 | **0.015** |  | | 2.26 | | 0.84, 3.69 | **0.002** |  | | 2.01 | | 0.67, 3.34 | **0.003** |  |
| Threonine | FFM (kg) |  | 0.048 | 0.000, 0.081 | **0.016** |  | | 0.043 | | 0.003, 0.082 | **0.035** |  | | 0.011 | | -0.027, 0.049 | 0.58 |  |
| Threonine | FFMI (kg/m^2^) |  | 0.048 | 0.000, 0.081 | **0.048** |  | | 0.046 | | 0.005, 0.086 | **0.028** |  | | 0.028 | | -0.013, 0.068 | 0.19 |  |
| Threonine | IGF-I (ng/ml) |  | 4.16 | 2.77, 5.56 | **<0.001** |  | | 3.72 | | 2.32, 5.13 | **<0.001** |  | | 2.79 | | 1.43, 4.15 | **<0.001** |  |
| Tryptophan | IGF-I (ng/ml) |  | 2.72 | 1.29, 4.15 | **<0.001** |  | | 2.40 | | 0.97, 3.82 | **0.001** |  | | 1.55 | | 0.17, 2.9..2 | **0.028** |  |
| Valine | FFM (kg) |  | 0.060 | 0.021, 0.099 | **0.003** |  | | 0.058 | | 0.018, 0.097 | **0.004** |  | | 0.022 | | -0.016, 0.060 | 0.26 |  |
| Valine | IGF-I (ng/ml) |  | 4.64 | 3.24, 6.03 | **<0.001** |  | | 4.38 | | 2.98, 5.77 | **<0.001** |  | | 3.23 | | 1.86, 4.60 | **<0.001** |  |
| Semi-essential |  |  |  |  |  |  | |  | |  |  |  | |  | |  |  |  |
| Arginine | IGF-I (ng/ml) |  | 3.23 | 1.80, 4.65 | **<0.001** |  | | 2.86 | | 1.43, 4.29 | **<0.001** |  | | 2.03 | | 0.65, 3.40 | **0.004** |  |
| L-glutamine | IGF-I (ng/ml) |  | 2.47 | 1.04, 3.89 | **<0.001** |  | | 2.09 | | 0.66, 3.52 | **0.004** |  | | 1.22 | | -0.15, 2.59 | 0.082 |  |
| Glycine | IGF-I (ng/ml) |  | 1.60 | 0.16, 3.03 | **0.030** |  | | 1.11 | | -0.33, 2.56 | 0.13 |  | | 1.52 | | 0.17, 2.87 | **0.027** |  |
| Proline | FFM (kg) |  | -0.051 | -0.090, -0.012 | **0.011** |  | | -0.056 | | -0.095, -0.017 | **0.005** |  | | -0.052 | | -0.089, -0.016 | **0.005** |  |
| Tyrosine | HAZ |  | 0.058 | 0.005, 0.11 | **0.033** |  | | 0.057 | | 0.004, 0.11 | **0.036** |  | | 0.020 | | -0.034, 0.074 | 0.46 |  |
| Tyrosine | FFM (kg) |  | 0.050 | 0.011, 0.089 | **0.012** |  | | 0.048 | | 0.009, 0.088 | **0.016** |  | | 0.010 | | -0.028 0.048 | 0.60 |  |
| Tyrosine | IGF-I (ng/ml) |  | 4.64 | 3.25, 6.03 | **<0.001** |  | | 4.52 | | 3.14, 5.91 | **<0.001** |  | | 3.27 | | 1.92, 4.63 | **<0.001** |  |
| Non-essential |  |  |  |  |  |  | |  | |  |  |  | |  | |  |  |  |
| L-asparagine | IGF-I (ng/ml) |  | 2.23 | 0.80, 3.63 | **0.002** |  | | 1.74 | | 0.32, 3.17 | **0.017** |  | | 1.43 | | 0.08, 2.77 | **0.038** |  |
| Glutamic acid | IGF-I (ng/ml) |  | 2.91 | 1.49, 4.32 | **<0.001** |  | | 2.51 | | 1.09, 3.93 | **<0.001** |  | | 2.54 | | 1.20, 3.87 | **<0.001** |  |
| Other |  |  |  |  |  |  | |  | |  |  |  | |  | |  |  |  |
| Citrulline | IGF-I (ng/ml) |  | 3.64 | 2.22, 5.05 | **<0.001** |  | | 3.07 | | 1.62, 4.52 | **<0.001** |  | | 2.16 | | 0.77, 3.54 | **0.002** |  |
| Hydroxy-L-proline | FFM (kg) |  | 0.061 | 0.022, 0.100 | **0.002** |  | | 0.055 | | 0.015, 0.095 | **0.007** |  | | 0.028 | | -0.009, 0.066 | 0.14 |  |
| Hydroxy-L-proline | IGF-I (ng/ml) |  | 4.04 | 2.64, 5.43 | **<0.001** |  | | 3.62 | | 2.21, 5.03 | **<0.001** |  | | 2.85 | | 1.50, 4.20 | **<0.001** |  |
| Ornithine | IGF-I (ng/ml) |  | 1.75 | 0.30, 3.21 | **0.018** |  | | 1.63 | | 0.19, 3.08 | **0.027** |  | | 1.72 | | 0.35, 3.09 | **0.014** |  |
| Taurine | HAZ |  | 0.071 | 0.018, 0.13 | **0.009** |  | | 0.065 | | 0.010, 0.12 | **0.021** |  | | 0.048 | | -0.005, 0.10 | 0.074 |  |
| Taurine | FFM (kg) |  | 0.047 | 0.008, 0.086 | **0.019** |  | | 0.040 | | 0.000, 0.080 | 0.052 |  | | 0.020 | | -0.018, 0.057 | 0.31 |  |
| Taurine | IGF-I (ng/ml) |  | 3.57 | 2.13, 5.01 | **<0.001** |  | | 3.08 | | 1.62, 4.54 | **<0.001** |  | | 2.68 | | 1.29, 4.07 | **<0.001** |  |
| Essential | FFM (kg) |  | 0.046 | 0.006, 0.085 | **0.024** |  | | 0.043 | | 0.003, 0.083 | **0.034** |  | | 0.014 | | -0.024, 0.053 | 0.47 |  |
| Essential | IGF-I (ng/ml) |  | 4.08 | 2.66, 5.51 | **<0.001** |  | | 3.80 | | 2.37, 5.22 | **<0.001** |  | | 2.89 | | 1.50, 4.27 | **<0.001** |  |
| Semi-essential | IGF-I (ng/ml) |  | 2.47 | 1.04, 3.89 | **<0.001** |  | | 2.05 | | 0.62, 3.49 | **0.005** |  | | 2.14 | | 0.80, 3.49 | **0.002** |  |
| Branched chain | FFM (kg) |  | 0.072 | 0.005, 0.14 | **0.036** |  | | 0.068 | | 0.00, 0.14 | **0.049** |  | | 0.017 | | -0.048, 0.082 | 0.61 |  |
| Branched chain | IGF-I (ng/ml) |  | 6.84 | 4.41, 9.26 | **<0.001** |  | | 6.38 | | 3.96, 8.79 | **<0.001** |  | | 4.76 | | 2.41, 7.10 | **<0.001** |  |

^1^Values are slope coefficients, 95%CI, p-values. *n=*699-730*.* ^b^ Model 1 is adjusted for age, sex and fasting time.
